# Modulation of ErbB2 Blockade in ErbB2-Positive Cancers: The Role of ErbB2 Mutations and PHLDA1

**DOI:** 10.1371/journal.pone.0106349

**Published:** 2014-09-19

**Authors:** Guangyuan Li, Xiaoqi Wang, Hanina Hibshoosh, Cheng Jin, Balazs Halmos

**Affiliations:** 1 Department of Pathology, University Hospitals of Case Medical Center, Case Western Reserve University, Cleveland, Ohio, United States of America; 2 Division of Hematology/Oncology, Herbert Irving Comprehensive Cancer Center, Columbia University Medical Center, New York, New York, United States of America; 3 Department of Pathology, Columbia University Medical Center, New York, New York, United States of America; Sun Yat-sen University Medical School, China

## Abstract

We set out to study the key effectors of resistance and sensitivity to ErbB2 tyrosine kinase inhibitors, such as lapatinib in ErbB2-positive breast and lung cancers. A cell-based *in vitro* site-directed mutagenesis lapatinib resistance model identified several mutations, including the gatekeeper ErbB2 mutation ErbB2-T798I, as mediating resistance. ErbB2-T798I engineered cell models indeed show resistance to lapatinib but remain sensitive to the irreversible EGFR/ErbB2 inhibitor, PD168393, suggestive of potential alternative treatment strategies to overcome resistance. Gene expression profiling studies identified a select group of downstream targets regulated by ErbB2 signaling and define PHLDA1 as an immediately downregulated gene upon oncogenic ErbB2 signaling inhibition. We find significant down-regulation of PHLDA1 in primary breast cancer and PHLDA1 is statistically significantly less expressed in ErbB2 negative compared with ErbB2 positive tumors consistent with its regulation by ErbB2. Lastly, PHLDA1 overexpression blocks AKT signaling, inhibits cell growth and enhances lapatinib sensitivity further supporting an important negative growth regulator function. Our findings suggest that PHLDA1 might have key inhibitory functions in ErbB2 driven lung and breast cancer cells and a better understanding of its functions might point at novel therapeutic options. In summary, our studies define novel ways of modulating sensitivity and resistance to ErbB2 inhibition in ErbB2-dependent cancers.

## Introduction

ErbB2 is a cell membrane surface-bound receptor tyrosine kinase and is involved in the signal transduction pathways leading to cell growth and proliferation. This gene is amplified in 10–20% of breast cancers, and amplification results in protein overexpression, which denotes an aggressive phenotype [1]–[4]. Overexpression, amplification and occasionally activating mutations of ErbB2 also occur in other cancers, including non-small cell lung cancer [5], [6]. The humanized antibody trastuzumab (Herceptin) against the overexpressed ErbB2 is proven to be effective in treating breast and gastric cancers with ErbB2 amplification [7]. Further, somatic mutations in the kinase domain of ErbB2 were discovered in various solid cancers including lung and breast carcinomas [8]–[12]. More recently, dual EGFR/ErbB2 tyrosine kinase inhibitors have also shown promise in clinical studies. A dual, reversible EGFR/ErbB2 inhibitor, lapatinib (Tykerb) in particular has demonstrated significant activity in ErbB2-positive breast cancers and now is approved to be used in this indication [13]. http://en.wikipedia.org/wiki/HER2/neu - cite_note-3Inhibition of ErbB2 tyrosine autophosphorylation by lapatinib abrogates downstream Ras-Raf-ERK1/2 and PI3K-AKT growth/survival signaling in ErbB2 overexpressing breast cancer cell lines and in patients with ErbB2-overexpressing breast cancers [14].

The uniform development of acquired resistance to lapatinib unfortunately limits its clinical efficacy. In analogous settings, secondary mutations, such as KIT exon 17 mutations in imatinib-resistant GIST and EGFR T790M in EGFR-mutated lung cancers are common mechanisms of resistance [8], [15]. There is no in vivo data currently available on potential mechanisms of resistance to lapatinib. In an in vitro model system, the secondary mutation, ErbB2 T798I imparts the strongest lapatinib resistance effect in Ba/F3 cells and is analogous to the epidermal growth factor receptor T790M [16]–[18]. A recent study suggests that in in vitro model systems the development of co-dependence on ER-signaling pathways might be another potential mechanism to lapatinib resistance [19]. There is also data to suggest the involvement of AXL activation in lapatinib-resistance in ErbB2 positive breast cancer [20]. Clearly, other mechanisms exist as well. Given the difficulties of obtaining primary patient specimens, it is important to construct experimental cellular systems to screen for relevant resistance mechanism that then permit testing of alternative inhibitors to overcome resistance. It is clearcut that the AKT/PI3K and ERK/MAPK pathways are key immediate downstream effectors of ErbB2 oncogenic signaling. However, their targeting has serious shortcomings in terms of toxicity and a narrow therapeutic index given their important physiological roles. Therefore, a better understanding of the entire array of downstream changes should allow the identification of novel actionable targets and the development of more effective and durable strategies for the treatment of ErbB2-positive cancers.

In our study, we set out to the dual goal to advance our understanding in both of these pivotal areas of oncogenic ErbB2 signaling- acquired resistance and downstream effector and modulator pathways. First, we established sensitivity to lapatinib treatment of ErbB2-positive lung and breast cancer cell lines, identified an array of putative acquired resistance mutations and demonstrated that mutations of ErbB2-T798 lead to resistance that can be overcome by the irreversible ErbB2 inhibitor, PD168393. Next, we completed a gene expression profiling study identifying a key group of early ErbB2 target genes and identify PHLDA1 as a novel downstream target of ErbB2 signaling with a functional role in negative feedback mechanisms to fine-tune the output of ErbB2 signaling and thereby significantly enhancing the sensitivity of ErbB2-positive breast cancer cells to lapatinib. Our studies provide several new avenues to extend the benefits of ErbB2 inhibition in the management of ErbB2-dependent cancers.

## Materials and Methods

### Patient Material

The breast cancer tissue and the normal breast tissue surrounding the tumor were obtained from same patient. The formalin-fixed tissues were used for immunohistochemistry study. The project was approved by the Ethics Committee of New York Presbyterian Hospital of Columbia University, and the patients gave written consent in accordance with the Declaration of Helsinki.

### Reagents

EGF was purchased from Sigma-Aldrich (St. Louis, MO), PD168393 and CL387,785 were purchased from Calbiochem (Billerica, MA), lapatinib was purchased from SELLECK Chemicals (Houston, TX). Drugs were dissolved in DMSO to give a 10 mmol/L stock solution and the final DMSO concentration in all experiments was <0.1%.

### Cell Culture

Calu3, HCC827, H1975, HCC202, HCC1569, HCC1937 and T47D cells were maintained in RPMI 1640 supplemented with 10% FBS, SkBr3 cells were maintained in McCoy's medium supplemented with 10% FBS. MDA-MB-468 and MDA-MB-436 cells were maintained in Leibovitz's L-15 medium supplemented with 10% FBS, MCF-7 cells were maintained in DMEM medium supplemented with 10% FBS. All cells were grown at 37°C in a humidified atmosphere with 5% CO_2_ and were in the logarithmic growth phase at the initiation of all experiments. MCF-7, MDA-MB-468 and MDA-MB-436 cells are provided here courtesy of Dr. Matthew Maurer (Columbia University). All cell lines were originally purchased from ATCC (Manassas, VA).

### ErbB2 mutagenesis, library generation, and drug resistance screen

The plasmid construct pcDNA3.1(-)-ErbB2-HA was generated as previously described [8]. We introduced a new enzyme site-AgeI at position 1457 in the ErbB2 cDNA. Then we shuffled the entire transmembrane and tyrosine kinase binding domain of ErbB2 by AgeI-AfeI digestion (AfeI site- position 3191). Using the mutated pcDNA3.1(-)-ErbB2-HA plasmid, we performed random mutagenesis using primers spanning the 1457-3191 region in the presence of 50 µmol/L MnCl_2_. Subcloning into p-GEM-T Easy vector and sequencing of individual colonies showed that essentially all of the PCR products contained mutations, with 50% of the products containing a 1-bp change. The pool of amplified DNA fragments was digested with AgeI and AfeI and religated into the backbone pcDNA3.1(-)-ErbB2-HA vector. Parental SkBr3 cells were transfected with the mutagenized libraries. Cell clones were selected by fresh medium containing 500 µg/mL G418 and 1 µmol/L lapatinib after 24-hour transfection. One month later, cell colonies were isolated and further amplified in the presence of both G418 and lapatinib and then the tyrosine kinase domain of ErbB2 was resequenced. Sequence alignment and analysis was done with DNASTAR and Mutation SURVEYOR software (SoftGenetics, State College, PA).

### Generation of the ErbB2 T798I plasmid construct

The ErbB2 T798I expression vector was generated by site-directed mutagenesis (QuickChange mutagenesis kit, Stratagene, Santa Clara, CA) of the pcDNA3.1(-)-ErbB2-HA plasmid construct. The primers used for site-directed mutagenesis were T798I, 5′-CACGGTGCAGCTGGTGATACAGCTTATGCCCTATG-3′ (sense) and 5′-CATAGGGCATAAGCTGTATCACCAGCTGCACCGTG-3′ (antisense). The proper sequence of the generated construct was confirmed by resequencing. We also constructed an ErbB2 wild type expression construct as the control for transfection assays. Transient transfection studies were done using Cos-7 cells with these reconstructed plasmids. Western blot analysis was used to confirm the success of transfection with the use of an anti-hemagglutinin tag antibody. G418 (500 µg/mL) was used to select stably transfected cells and isolated colonies were picked using cloning rings and continued to be cultured in the presence of G418. Expression of the ErbB2 T798I mutant protein was confirmed by the detection of the expression of the hemagglutinin tag as above.

### Cell Growth Inhibition Analysis

Cells were plated in each well of 96-well plates in cell medium containing 10% FBS. 24 hours after plating, cells were treated with specified concentrations of inhibitors. The concentration of DMSO in the medium was adjusted to 0.1%. After 72 hours of incubation, viable cell numbers were measured by using 3-(4,5-dimethylthiazol-2-yl)-5-(3-carboxymethoxyphenyl)-2-(4-sulfophenyl)-2*H*-tetrazolium, inner salt (MTS; absorption of formazan at 490 nm; CellTiter96, Promega, Madison, WI) according to the manufacturer's protocol. Each assay consisted of three replicate wells of the same drug concentration. The IC50 was determined from the dose-response curves.

### Immunoblotting

To assess ErbB2 signaling activation, cells were starved for 12 hours and then incubated with lapatinib or PD168393 for 3 hours followed by EGF (100 ng/ml) or heregulin (20 ng/ml) stimulation for 15 minutes. Whole cell lysates were separated on 8% SDS-polyacrylamide gels, transferred to nitrocellulose, and protein expression was detected by the use of Western Lightning Chemiluminescence Reagent (Perkin-Elmer Life Science, Wellesley, MA). Phospho-Akt (Ser473), phospho-ERK 1/2 (T202/Y204), total-Akt, total-ERK and GAPDH antibodies were purchased from Cell Signaling Technology (Beverly, MA). Antibodies to ErbB2 (C-18), PHLDA1 [21]-[23] and hemagglutinin tag (F-7) were purchased from Santa Cruz Biotechnology (Santa Cruz, CA). Antibody directed against phospho-ErbB2 (Y1248) was purchased from R&D systems (Minneapolis, MN). Secondary anti-mouse and anti-rabbit antibodies were purchased from Santa Cruz Biotechnology. Experiments were repeated three times.

### Annexin/propidium iodide and BrdU assays

For both assays, samples were analyzed on a fluorescence-activated cell scan cytometer EPICS XL MCL (Beckman Coulter, Miami, FL). Annexin/propidium iodide (PI) apoptosis assay was performed according to the manufacturer's instructions (Annexin V-FLUOS staining kit, Roche Applied Science, Indianapolis, IN). Cells were seeded at 1×10^6^ cells/well on 10 cm plates; 24 hours after plating media was refreshed to specified concentrations of inhibitors, then incubated for an additional 72 hours. Cells were then trypsinized, washed with PBS, resuspended in Annexin/Propidium iodide staining buffer (Roche Applied Science), and then analyzed by flow cytometry. Data were analyzed with FlowJo software (Tree Star, Ashland, OR). Bromo-deoxyuridine (BrdU) cell proliferation assay was performed according to the manufacturer's instructions (FITC BrdU Flow Kit, BD Pharmingen, San Diego, CA). Briefly, Calu3 cells were treated with lapatinib at different concentrations (final concentration: 0, 0.1, 1, 3, and 10 µmol/L) for 3 days, then pulse labeled for 60 min with 10 µM BrdU, collected by trypsinization and permeabilized with BD cytoperm buffer, stained with fluorescent anti-BrdU antibody, counterstained with 7-amino-actinomycin D for total DNA content and analyzed by flow cytometry. Experiments were repeated at least three times.

### Oligonucleotide Array Analysis

Calu3 and SkBr3 cells were grown to 60% confluence in culture medium and then were treated with lapatinib (1 µm), or DMSO (0.01%) as a control for 6 hours. Total cellular RNA was isolated using the RNAeasy mini kit (Qiagen, Germantown, MD). RNA quality was assessed using Experion Automated Electrophoresis Station (Bio-Rad) and quantified by fiberoptic spectrophotometry using the Nanodrop ND-1000 (Nanodrop Inc., Wilmington, DE, USA). cDNA was synthesized, labeled and hybridized to Affymetrix HG-U133A microarrays and scanned. The expression value for each gene was calculated using Affymetrix GeneChip software and the Robust Multichip Average (RMA) method for signal extraction.

### Preprocessing, Filtering, and Statistical analysis

The microarray data were analyzed and the false discovery rate was calculated using SAM (Stanford University, CA). Array normalization and computation of expression values of the chip were performed using DNA-Chip Analyzer (dchip). For annotation of the resulting genes, the gene ontology browser NetAffymetrix (http://ww.affymetrix.com) was used. The differentially expressed genes with ≥2 or ≤0.5 fold change, q-value ≤1% were analyzed using t test and clustered with the software package cluster 3.0 [24].

### Quantitative PCR Assay

Total RNA from triplicate samples was isolated by using RNAeasy mini kit (Qiagen). cDNA was synthesized with M-MLV reverse transcriptase (SuperScipt III reverse transcriptase, Invitrogen, Grand Island, NY) with the use of oligo(dT) primers from Invitrogen. All samples were analyzed by Roche LightCycler using the Syber green probes. Primer sequences are shown in Table S1. Levels of GAPDH expression were used as internal references to normalize input cDNA. Ratios of level of each gene to GAPDH were then calculated.

### Immunohistochemistry

Formalin-fixed primary breast tumor tissue sections were deparaffinized and rehydrated and incubated with 0.6% hydrogen peroxide in methanol. Target retrieval solution, pH 9.0 (Dako, Carpinteria, CA) was used for antigen retrieval. The slides were incubated with primary antibodies for overnight at 4°C followed by staining using the R.T.U Vectastain Universal Quick Kit (Vector Laboratories, Burlingame, CA) with hematoxylin nuclear counterstaining. Finally, slides were dehydrated sequentially in ethanol, cleared with xylenes. The anti-PHLDA1 antibody was used in 1∶50 dilution (Santa Cruz Biotechnology). The intensity of the staining of PHLDA1 was scored by a board certified breast cancer pathologist as 0 (no expression), 1+ (weak expression), 2+ (moderate expression) and 3+ (strong expression) and the percentage (0–1.0) of positively stained tumor cells was also evaluated to generate a composite H-score (intensity score X percent positive, range of possible values 0–3.0). Silver in situ hybridization (SISH) was used to determine ErbB2 status in human breast cancer samples [25]. The ratio of ErbB2/chromosome 17 was calculated by dividing the total score for ErbB2 by the total score for chromosome 17. A ratio of <1.8 indicated that ErbB2 gene was not amplified, whereas a ratio of >2.2 indicated amplification of the gene. For cases with a ratio between 1.8 and 2.2, signals from 20 more tumor nuclei were counted in each slide and a new ratio was calculated.

### Plasmid constructs and cellular transfection

Full length cDNA sequence of human PHLDA1 was amplified from genomic DNA of HCC827 cells. PHLDA1 was then modified with an HA-tag at the C-terminus by overlapping PCR to distinguish plasmid derived from native protein and was subcloned into the pcDNA3.1(-) backbone vector. The accuracy of the construct was confirmed by direct DNA sequencing. SkBr3 cells were transiently transfected with empty pcDNA3.1(-) (pcDNA3.1(-)-EV) or PHLDA1 (pcDNA3.1(-)-PHLDA1) expression vectors using Fugene 6 (Roche Applied Science). Stably transfected subclones were selected with G418 at a concentration of 500 µg/ml starting 48 h posttransfection.

### Anchorage independent growth assay and migration assay

For anchorage independent growth assay, pcDNA3.1(-)-EV and pcDNA3.1(-)-PHLDA1 stable transfected cells were trypsinized, counted twice using a hemocytometer, and plated at 5000 cells/ml in top plugs consisting of 0.4% SeaPlaque agarose (FMC Bioproducts, Rockland, ME) in McCoy's 5A medium. After 25 days, the colonies were photographed and counted. The experiment was repeated three times with triplicates each. Average numbers of colonies from each experiment were plotted. For migration assays, confluent monolayers of pcDNA3.1(-)-EV and pcDNA3.1(-)-PHLDA1 stable transfected cells were scratched with a sterile 10 µl pipette tip. The plates were then washed and incubated at 37°C in 10%FBS/McCoy's 5A medium for 48 hours.

### Clonogenic survival assay

Two thousand pcDNA3.1(-)-EV and pcDNA3.1(-)-PHLDA1 SkBr3 cells were plated onto 6 cm plates and were allowed to recover for 24 hours. Cells were then treated with 0.1 µM or 0.5 µM lapatinib and were allowed to grow for 10 days. Next, the plates were stained with Methylene Blue for 4 hours and the number of colonies with more than 50 cells in each plate were determined. A set of untreated plates were included as controls to determine the plating efficiency for individual cell lines. The effective number of cells/plate was determined on the basis of the plating efficiency of each cell line and the number of cells plated per plate. These numbers were then used to calculate the percentage survival. Also, each treatment was performed in triplicates in order to determine the SD for each data point.

### Statistical analysis

Statistical analyses were performed using chi-squared test or two-sample *t*-tests as appropriate, with *P*-value of <0.05 considered statistically significant. Data are expressed as mean ± SD. The data are representative of three separate experiments.

## Results

### Lapatinib and PD168393 induce growth inhibition and apoptosis in ErbB2-positive Calu3 and SkBr3 cells

**Figure 1:**
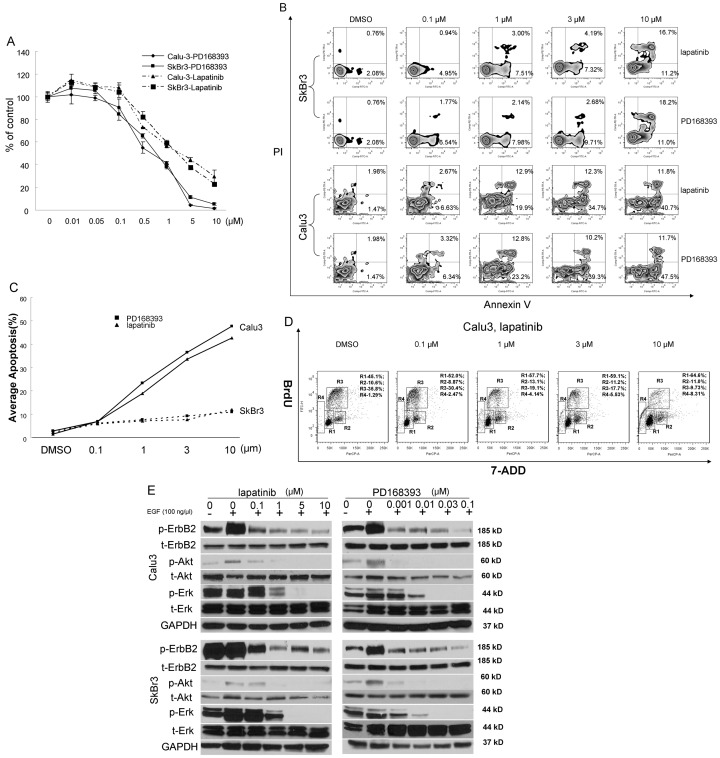
Antitumor effects of lapatinib and PD168393 in the ErbB2 positive lung cancer cell line, Calu3 and the ErbB2 positive breast cancer cell line, SkBr3 after 72 hour treatment. A. Cellular proliferation of Calu3 and SkBr3 cells treated with different concentrations of lapatinib and PD168393 as determined by the MTS assay. B. Lapatinib and PD168393 induce cellular apoptosis in Calu3 and SkBr3 cells. Representative Annexin V/propidium iodide flow cytometry; the numbers represent percentage of cells in the appropriate quadrant. C. Quantification of apoptosis. D. Lapatinib induces cell cycle arrest in Calu3 cells as detected by BrdU and 7-ADD assay. R1-G1 phase; R2-G2 phase; R3-S phase; R4-G0 phase. E. Dose response of lapatinib and PD168393 on phosphorylation of ErbB2, AKT and ERK in Calu3 and SkBr3 cells in response to EGF treatment. p-ErbB2: phosphorylated ErbB2; t-ErbB2: total-ErbB2; p-AKT: phosphorylated AKT; t-AKT: total AKT; p-ERK: phosphorylated ERK; t-ERK: total ERK.

We first conducted MTS cell growth assays to examine whether inhibition of ErbB2 by lapatinib, a reversible dual EGFR/ErbB2 tyrosine kinase inhibitor, or PD168393, which irreversibly inhibits ErbB2, can lead to growth inhibition of ErbB2-positive cancer cells, such as ErbB2-positive Calu3 lung cancer and SkBr-3 breast cancer cell lines. We found that both lapatinib and PD168393 significantly inhibited the growth of Calu3 and SkBr3 cells (Fig. 1A). Further investigation using Annexin V staining showed that both lapatinib and PD168393 induced prominent apoptosis in both Calu3 and SkBr3 cells (Fig. 1B and 1C). BrdU incorporation analysis was performed to show the reduced proliferation of these two cell lines by lapatinib treatment. The lapatinib treated Calu3 cells showed a significantly higher G_1_ population consistent with G_1_ arrest compared with control cells (Fig. 1D) and this was further confirmed in SkBr3 cells (Figure S1). These results suggest that lapatinib induces cell cycle arrest in the G_1_ phase and subsequent apoptosis in Calu3 and SkBr3 cells. In order to determine the effect on downstream signaling pathways, the phosphorylation levels of the MAP kinase and AKT pathways were assessed in these two ErbB2-positive cell models, and robust blockade of both signaling pathways was found (Fig. 1E, Figure S2). This finding was correlated with the results of the growth inhibition assays done, suggesting that the growth inhibition caused by lapatinib and PD168393 may be regulated through effective blockade of the MAP kinase and AKT pathways.

### Characterization of resistance mutant ErbB2-T798I

Next, we pursued a random mutagenesis strategy in order to assess secondary mutations of ErbB2 that can lead to functional resistance to lapatinib. First, the entire transmembrane and tyrosine kinase domain of ErbB2 (positions 1457–3191) was randomly mutagenized. Then, the mutagenized ErbB2 was reshuffled into a pcDNA3.1(-)-ErbB2-HA backbone and transfected into SkBr-3 cells. We generated drug-resistant SkBr-3 cell clones in the presence of both 500 µg/mL G418 and 1 µmol/L lapatinib and identified 9 distinct amino acid substitutions affecting 5 residues from isolated clonal derivatives. Substitutions at five positions were identified: P944L (3 cases), T798I (2 cases), T798M (1 case), C576S (1 case), R487P (1 case), and E542G (1 case). The critical gatekeeper threonine residue in the case of ErbB2 is T798- which corresponds to T790 of EGFR as well as T315 of the ABL kinase. Based on the nucleotide sequence of ErbB2, it is significantly more likely that a mutation would alter the sequence to T798I (requires one base pair change) versus T798M (requires two base pair changes). Therefore, T798I was chosen for further studies.

**Figure 2:**
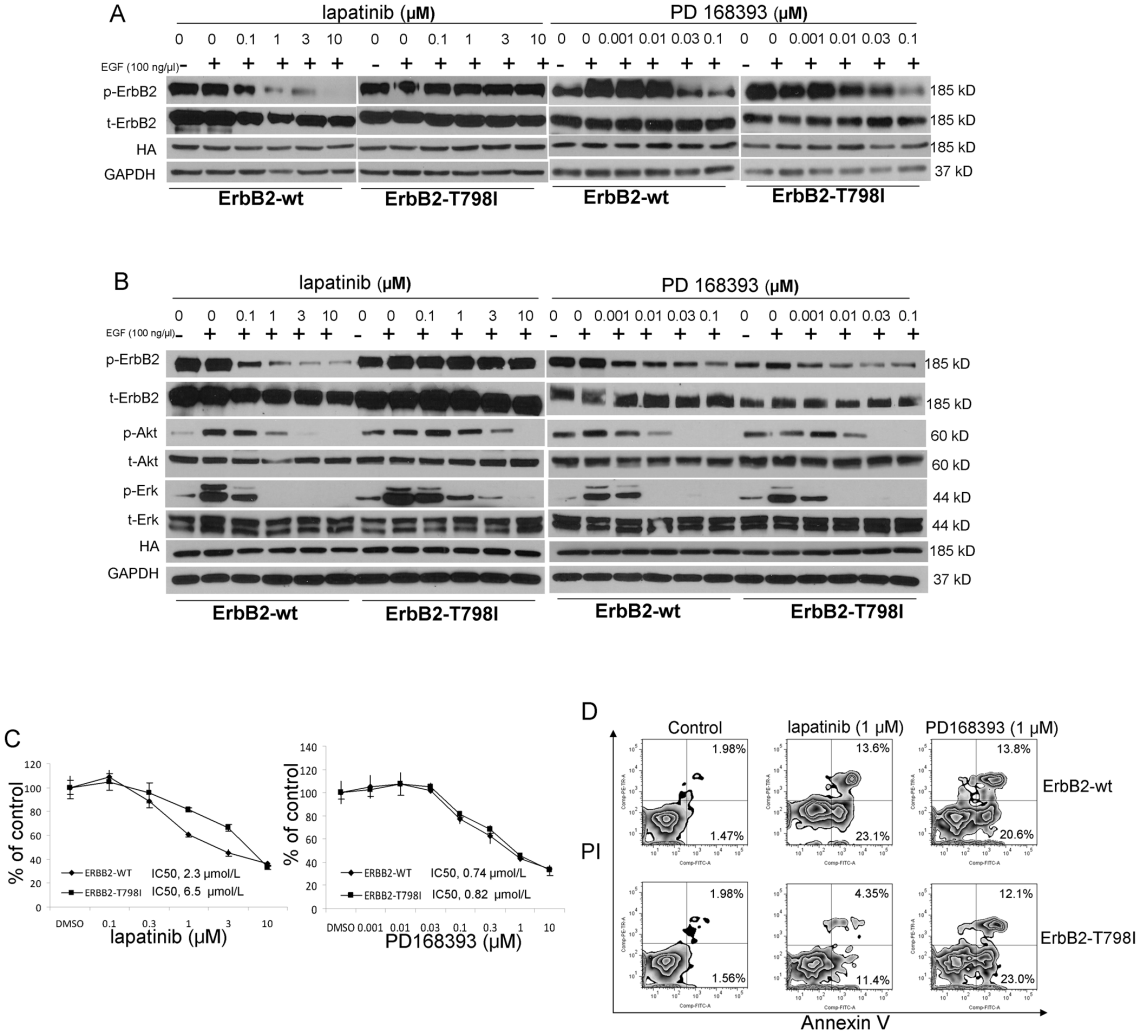
The irreversible inhibitor PD168393 overcomes lapatinib resistance caused by the ErbB2 T798I mutation. A. Dose response of lapatinib and PD168393 on phophorylation of ErbB2 in Cos7 cells after 24 hours of transient transfection with ErbB2-wt and ErbB2-T798I vectors. Total ErbB2, HA and GAPDH expression level as control. B. Dose response of lapatinib and PD168393 on phosphorylation of ErbB2, AKT and ERK in ErbB2-wt and ErbB2-T798I stable Calu3 cell clones. Total ErbB2, total AKT, total ERK, HA and GAPDH expression level as control. C. Dose-dependent growth inhibition induced by the treatment of lapatinib and PD168393 for 72 hours in ErbB2-wt and ErbB2-T798I stable Calu3 cell clones as detected by the MTS assay. D. Lapatinib and PD168393 induce cellular apoptosis in ErbB2-wt and ErbB2-T798I stable Calu3 cell clones after 72 hour treatment. E. Quantification of apoptosis.

T798I was regenerated by site-directed mutagenesis and used for transient transfection studies in Cos-7 cells. In pcDNA3.1(-)-ErbB2-wt Cos7 cells, 1 µmol/L lapatinib significantly inhibited phosphorylation of ErbB2. In contrast, in pcDNA3.1(-)-ErbB2-T798I transfectants, persistent ErbB2 phosphorylation was noted in the presence of lapatinib at concentrations ranging from 0.1 to 10 µmol/L, confirming the shift in drug sensitivity corresponding to the findings of the MTS assays (Fig. 2A). To confirm this observation, we also generated a stably transfected Calu3 cell clone with the pcDNA3.1(-)-ErbB2-T798I-HA plasmid construct and found very consistent result (Fig. 2B). We assessed the level of resistance that pcDNA3.1(-)-ErbB2-T798I conferred to lapatinib by generating dose-response curves by MTS assays on pcDNA3.1(-)-ErbB2-T798I stable transfectants. Cells were cultured in the presence of increasing concentrations of lapatinib ranging from 0 to 10 µmol/L. pcDNA3.1(-)-ErbB2-wt Calu3 cells were potently inhibited by lapatinib (IC_50_, 2.3 µmol/L). Calu3 cells expressing pcDNA3.1(-)-ErbB2-T798I were less sensitive to lapatinib with an IC_50_, 6.5 µmol/L (Fig. 2C).

We further analyzed the functional consequences of T798I by assessing cellular apoptosis induced by lapatinib as defined by Annexin/propidium iodide apoptosis assays. Treatment of pcDNA3.1(-)-ErbB2-wt Calu3 cells with 1 µmol/L of lapatinib resulted in a prominent increase in the percentage of apoptotic cells, whereas the same concentrations had induced significantly less apoptosis in pcDNA3.1(-)-ErbB2-T798I stable transfectants (Fig. 2D and 2E). These results were consistent with transient transfection studies pursued in SkBr3 cells (Figure S3, S4 and S5).

### An alternative ErbB2 inhibitor PD168393 can overcome resistance

Next, we tested the T798I Calu3 clones against the irreversible ErbB2/EGFR inhibitor PD168393. We carried out standard MTS assays following 72 hours of drug treatment using a range of concentrations from 0 to 10 µmol/L. Our data showed that PD168393 could overcome T798I induced resistance against lapatinib (Fig. 2C). This inhibitor was as effective against pcDNA3.1(-)-ErbB2-T798I as against pcDNA3.1(-)-ErbB2-wt transfectants. In signaling studies, we found that PD168393 completely inhibits AKT and ERK phosphorylation at concentrations as low as 0.03 µmol/L so that in order to better show the downstream signaling change, we used a range of concentrations from 0 to 0.1 µmol/L. Signaling studies showed that treatment with PD168393 can potently inhibit downstream signaling correlates such as phospho-AKT and ERK (Fig. 2B). ErbB2 phosphorylation level in cells with mutant ErbB2 was altered but to a lesser degree by PD168393 inhibition suggestive of partial inhibition (Fig. 2B). Annexin/propidium iodide studies further showed the induction of apoptosis by this compound in both pcDNA3.1(-)-ErbB2-wt and pcDNA3.1(-)-ErbB2-T798I Calu3 cell clones (Fig. 2D and 2E). These results suggest that T798I might be an important resistance mechanism in lapatinib-treated tumors but also suggest potential efficacy of irreversible ErbB2 inhibitors.

### Gene expression profiling of lapatinib-treated cells

Next, we set out to study a comprehensive array of downstream changes as a result of effective ErbB2 inhibition. Previously, in similar studies we have shown that as early as at 6 hours of signaling inhibition, the irreversible EGFR inhibitor, CL-387,785 could induce a significant change of transcription in a small and representative group of key downstream effectors of oncogenic EGFR signaling, including important pathway components such as Cyclin D1, DUSPs etc in EGFR mutant lung cancers [1], [26], [27]. In an analogous fashion, we set out to identify the early effectors of ErbB2 induced gene expression changes in ErbB2-positive cells. To identify the key genes differentially expressed in SkBr3 cells treated with lapatinib (1 µmol/L) for 6 hours, a gene expression profiling study was done in triplicates on total cellular RNA using Affymetrix HG-U133A chips. The raw expression data were preprocessed, rescaled, filtered, and analyzed as described in Materials and Methods. The genes altered by lapatinib treatment are shown in Table S2. This analysis showed that 110 marker genes were down-regulated and 73 genes were up-regulated with the strict criteria set of an at least 2 fold change and Delta value >5 when lapatinib-treated samples were compared with vehicle-treated cells (the Delta value is a tuning parameter that can dynamically change thresholds for significance).

**Figure 3:**
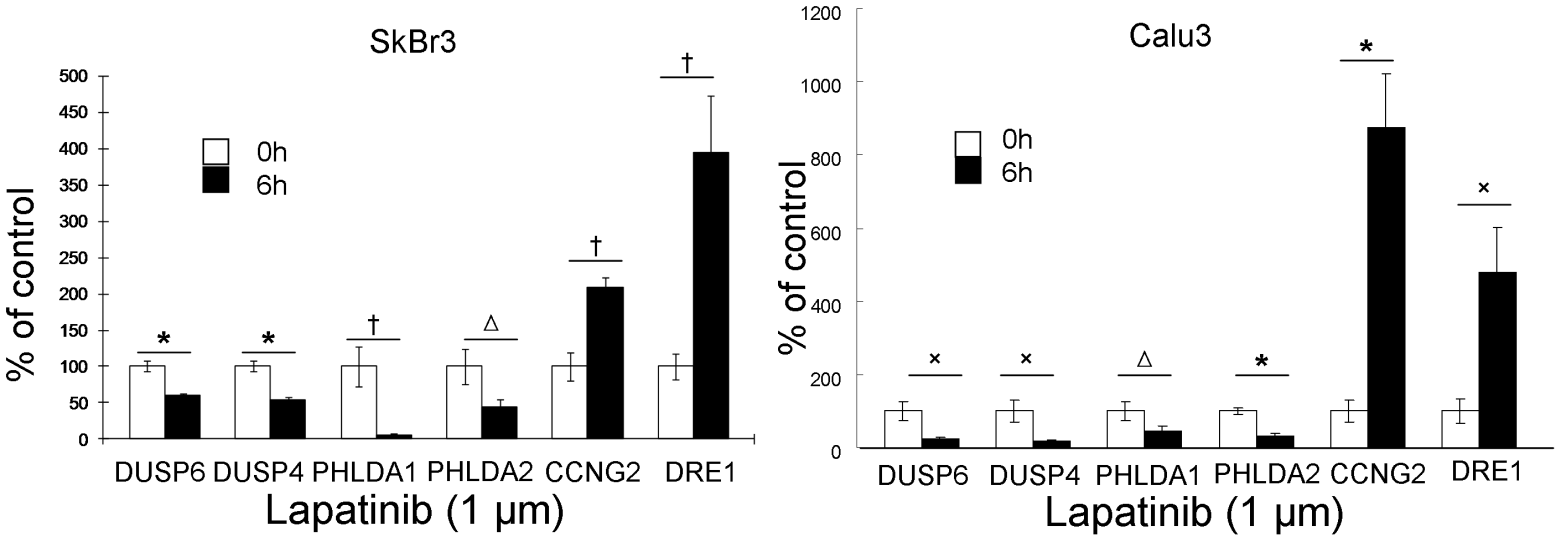
PHLDA1 is regulated by ErbB2 inhibition at the mRNA level at 6 hours. Quantitative PCR analysis of gene regulation by lapatinib treatment for 6 hours in SkBr3 and Calu3 cells. Fold induction relative to 0 hour control was plotted after normalization by GAPDH. Δ: p<0.05;×: p<0.01; †: p<0.005; *: p<0.001.

A comparison of this gene list with our previous data identifying gene changes upon EGFR inhibition in EGFR-dependent lung cancer cells [27], [28] showed a remarkable overlap between the significantly modulated genes in the two independent experiments (Table 1). More than half of the genes identified in our previous study of gene regulation by EGFR blockade were significantly modulated upon ErbB2 inhibition in ErbB2-positive cancer cells as well suggesting highly shared oncogenic pathways. Six of the top ErbB2/EGFR-regulated genes, DUSP6, DUSP4, PHLDA1, PHLDA2, CCNG2 and DRE1 were selected and confirmed to be strongly regulated by quantitative reverse transcription-PCR analysis on RNA obtained from lapatinib-treated cells (Fig. 3).

**Table 1:** List of the overlapping regulated genes between ErbB2 blockade by lapatinib in SkBr3 cells and EGFR blockade by CL-387,785 in H1975 cells.

Gene ID	ErbB2 blockade Fold Change	EGFR blockade Fold Change	Gene Name
221986_s_at	4.95483	2.78	KLHL24
212675_s_at	2.903494	2.01	KIAA0582 protein
202769_at	2.013582	3.12	CCNG2
201631_s_at	0.304776	0.45	IER3
209803_s_at	0.125931	0.47	PHLDA2
209457_at	0.460962	0.45	DUSP5
208892_s_at	0.126178	0.24	DUSP6
201464_x_at	0.491798	0.43	JUN
204014_at	0.38073	0.27	DUSP4
203499_at	0.125657	0.47	EPHA2
204420_at	0.112585	0.33	FOSL1
208712_at	0.150877	0.42	CCND1
204363_at	0.311877	0.34	F3

### PHLDA1 is down-regulated by EGFR/ErbB2 inhibition

**Figure 4:**
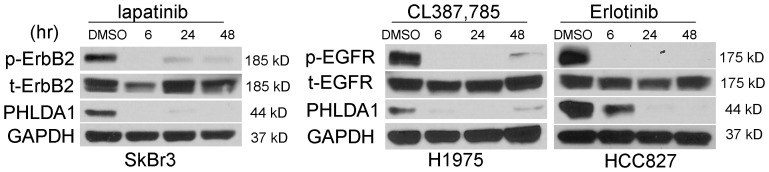
Downregulation of PHLDA1 expression upon ErbB2/EGFR inhibition. Western blot analysis following treatment with lapatinib, erlotinib and CL387,785 at 1 µm for 6, 24 and 48 hours in ErbB2-positive SkBr3 and EGFR-mutated H1975, HCC827. DMSO treatment was 48 hours. Different cell lines were used with the relevant inhibitor capable of blockade of oncogenic signaling.

We then decided to focus on the novel downstream target gene, PHLDA1 as a protein that previously was not identified to participate in ErbB2/EGFR driven pathways. This gene belongs to the group of pleckstrin-homology domain proteins and is speculated to function through interfering with the function of PI3-kinase thereby inhibiting AKT activation based on work on another family member, PHLDA3 [29]. Western blotting analysis indeed showed that PHLDA1 was down-regulated significantly as early as 6 hours into EGFR or ErbB2 inhibition on the protein level as well that represents an immediate response (Fig. 4). These results suggest that PHLDA1 is immediately and significantly down-regulated by suppression of EGFR/ErbB2 signaling at both mRNA and protein levels.

### PHLDA1 is down-regulated in human breast cancers

**Figure 5:**
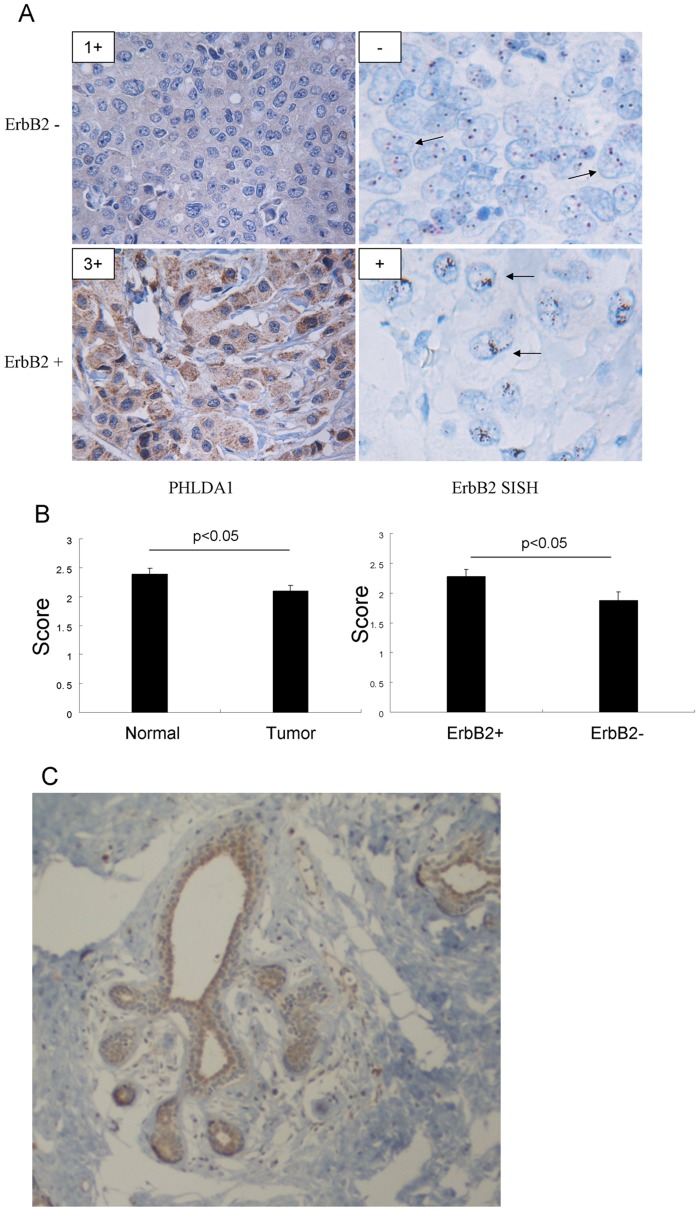
PHLDA1 is down-regulated in human breast cancers. A. Immunohistochemical staining of PHLDA1 in 24 primary breast cancers. The intensity of the staining of PHLDA1 was scored as 0 (no expression), 1+ (weak expression), 2+ (moderate expression) and 3+ (strong expression). SISH was used to determine ErbB2 status in human breast cancer samples. B. Statistical analysis of IHC study. C, PHLDA1 is strongly expressed in normal breast epithelium.

We developed an IHC protocol for assessing expression of PHLDA1 and performed IHC staining of whole tissue sections on 24 primary breast cancer cases including 12 ErbB2 positive and 12 ErbB2 negative breast adenocarcinomas tested by SISH study and used available normal mammary tissues on the same slides as the internal normal control. SISH was used to determine ErbB2 status in human breast cancer samples. Scoring was performed by an expert breast pathologist and an H-score was defined for all samples (Fig. 5A). PHLDA1 expression was significantly reduced in human breast cancer tissue versus adjacent normal mammary epithelium (p<0.05) (Fig. 5B and 5C). The average score of staining in tumor tissue was 2.091±0.479 (mean±SD), while in the normal control it was 2.386±0.461 (mean±SD). Comparing the ErbB2 positive with ErbB2 negative samples, PHLDA1 was found to be significantly less expressed in ErbB2 negative tumor slides (p<0.05). Of the 12 ErbB2 positive slides, the average score of staining was 2.273±0.41, while of the 12 ErbB2 negative slides, the average score of staining was 1.875±0.483 (Table 2). This suggests robust expression of PHLDA1 in mammary epithelium, downregulation of PHLDA1 in tumor versus adjacent normal tissue suggestive of a putative tumor suppressor role and also regulation by ErbB2 as suggested by the profiling studies as well. We did not observe a significant correlation of PHLDA1 expression with ER status. We further determined the expression of PHLDA1 by Western blotting in ErbB2 positive (HCC202 and HCC1569) versus ErbB2 negative cell lines (MCF-7, T47D, MDA-MB-436, MDA-MB-468 and HCC1937). All ErbB2-positive cell lines express PHLDA1 while expression is variable in ErbB2-negative cell lines. In these cell lines PHLDA1 expression might be regulated by multiple upstream pathways converging to activate PHLDA1 expression besides ErbB2 (Figure S6).

**Table 2:** Description of patient population of immunohistochemistry.

	ErbB2+	ErbB2-	P value
ER			NS
Positive	6/12	9/12	
Negative	6/12	3/12	
PR			NS
Positive	6/12	8/12	
Negative	6/12	4/12	
Type			NS
IDC	11/12	10/12	
ILC	1/12	2/12	
Age	49.5±13.0	58.7±21.3	NS
PHLDA1 expression	2.273±0.41	1.875±0.483	p<0.05

NS, not significant; IDC, invasive ductal carcinoma;

ILC, invasive lobular carcinoma.

### PHLDA1 inhibits breast tumor cell proliferation and migration

**Figure 6:**
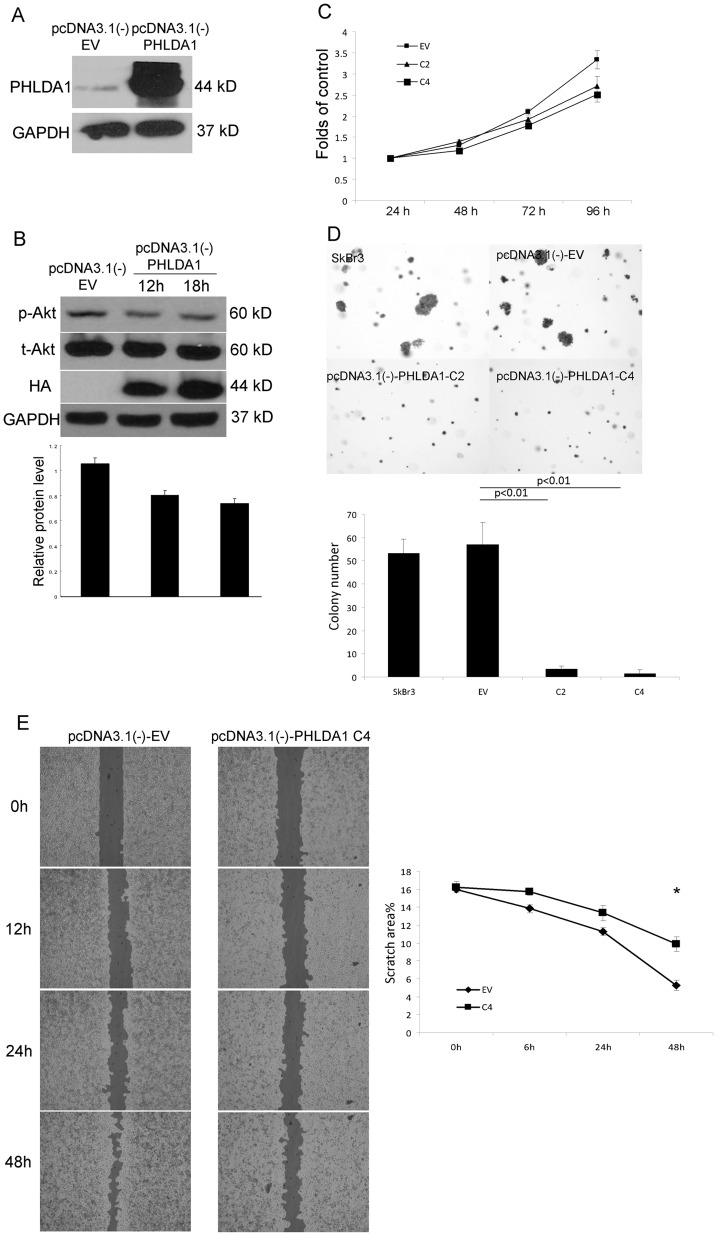
Tumor suppressor activity of PHLDA1 in vitro. A. Western blotting of whole cell lysates with anti-PHLDA1 shows strong expression of PHLDA1 in pcDNA3.1(-)-PHLDA1 transfected cells. B. Western blotting of cell lysates with pcDNA3.1(-)-PHLDA1 transient transfection shows potent inhibition of AKT phosphorylation. C. MTS cell growth assay of pcDNA3.1(-)-EV and pcDNA3.1(-)-PHLDA1 stable clones. D. Soft Agar assay for anchorage-independent cell growth is done with pcDNA3.1(-)-EV and pcDNA3.1(-)-PHLDA1 stable cell clones. E. Wound-healing assay shows that PHLDA1 overexpression significantly decreased cell mobility. *: p<0.05.

In order to further dissect the role of PHLDA1 in breast cancer cells, PHLDA1 expression constructs were generated and expression of PHLDA1 was confirmed by detection of the hemagglutinin tag (Fig. 6A). As early as 12 hours following transient transfection of SkBr3 cells, PHLDA1 significantly inhibited phosphorylation of AKT (Fig. 6B). Two PHLDA1 stable transfectants were selected in SkBr3 cells as C2 and C4 clones and cell proliferation was determined by MTS assay. PHLDA1 overexpression significantly reduced cell growth at 96 hours compared with pcDNA3.1(-)-EV stable transfectants in SkBr3 cells (Fig. 6C). The effect of PHLDA1 was further evaluated under anchorage-independent conditions. The results showed that PHLDA1 overexpression resulted in a dramatic loss in colony-forming ability (Fig. 6D). Thus, we showed that PHLDA1 upregulation inhibits cell proliferation under attached, as well as anchorage-independent conditions in breast cancer cells. Next, cell motility was determined by wound-healing assays in control and PHLDA1 overexpressing cells. PHLDA1 overexpression significantly decreased cell mobility, suggesting that it negatively regulates chemotaxis (Fig. 6E). These results suggest a strong growth-inhibitory effect of PHLDA1 in ErbB2-positive cancer cells, likely via negative feedback regulation of AKT-driven pathways.

### PHLDA1 significantly enhances the sensitivity of ErbB2-positive breast cancer cells to lapatinib

**Figure 7:**
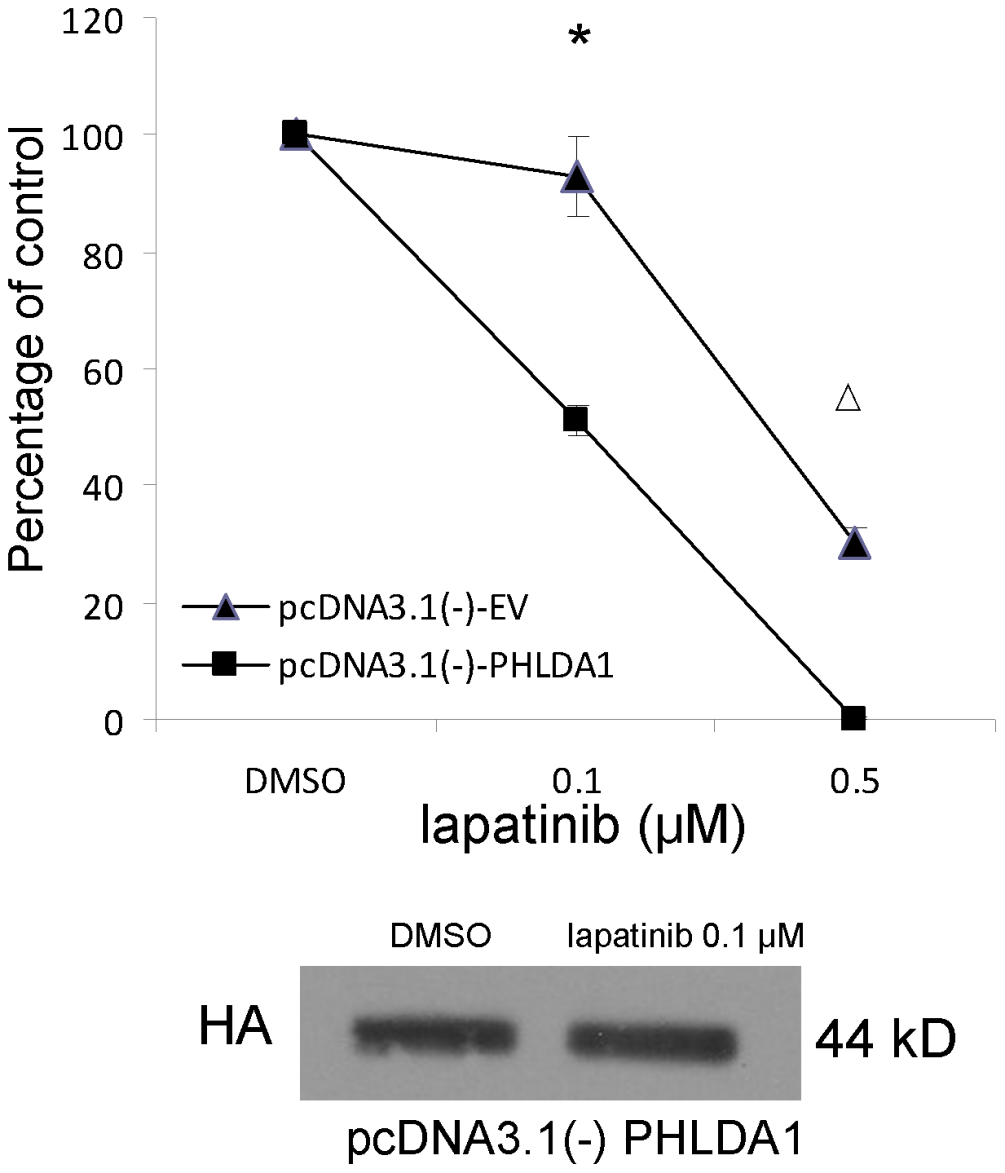
Clonogenic survival assay shows that PHLDA1 overexpression could significantly enhance lapatinib sensitivity in breast cancer cells. *: p<0.001; Δ: p<0.0001. Western blotting of cell lysates with anti-HA shows equal exogenous expression of PHLDA1 in pcDNA3.1(-)-PHLDA1 transfected cells after 10 days of treatment with DMSO and 0.1 um lapatinib.

A clonogenic survival experiment was used to assess the role of PHLDA1 in lapatinib sensitivity. The result of this experiment showed that SkBr3 cells with forced overexpression of PHLDA1 were significantly more sensitive to lapatinib than cells transfected with empty vector control. As shown in Figure 7, 0.1 µmol lapatinib inhibits colony formation by 8% in empty vector transfected cells while inhibits colony formation by 50% in PHLDA1-transfected cells. These data indicate that PHLDA1 expression can increase lapatinib sensitivity in breast cancer cells.

## Discussion

We set out to define novel ways to overcome resistance to lapatinib and understand downstream pathways of oncogenic ErbB2 signaling in ErbB2-dependent breast and non-small cell lung cancer cells. We showed as expected that both lapatinib as well as the irreversible ErbB2 inhibitor, PD168393 could induce apoptosis and inhibit cell growth in ErbB2 positive lung and breast cancer cell lines. This was correlated with a dose-dependent reduction of phosphorylation of ErbB2 and its downstream effectors, AKT and ERK1/2. In a random mutagenesis-based resistance screen, a number of potential secondary ErbB2 mutations, including mutations of the gatekeeper T798 residue were identified that might mediate acquired lapatinib-resistance. T798I is analogous to the common gatekeeper mutations of EGFR and BCR-ABL, EGFR-T790M and BCR-ABL T315I. We then further showed the functional relevance of the T798I ErbB2 mutation as a potential resistance mechanism against reversible ErbB2 inhibitors, modeled by lapatinib, in ErbB2-positive cancer cells. Indeed, T798I was confirmed to lead to functional resistance to lapatinib with an about 3-fold increase in the IC50 of lapatinib. As many imatinib-resistant mutations of BCR-ABL as well as the recently identified D761Y mutation of EGFR lead to an IC50 shift in this range, we predict that these mutations will be clinically relevant. Our studies suggest that T798I-induced lapatinib resistance can be overcome by irreversible dual ErbB2/EGFR inhibitors, such as PD168393. Analogous irreversible inhibitors, such as HKI-272 and BIBW2992, are being actively developed in this setting. Preclinical results show that BIBW2992 is effective in lung cancer models, including those with EGF receptor (EGFR) mutations resistant to reversible first-generation EGFR inhibitors. Epidermal growth factor receptor/ErbB2/vascular endothelial growth factor receptor inhibitor EXEL-7647 was found to inhibit almost all lapatinib resistance-associated mutations [16]. Rexer B et al found that simultaneous blockade of HER2 and EGFR could be an effective treatment strategy against HER2 gene-amplified breast cancer cells harboring T798M mutant alleles [18]. Our studies similarly suggest that irreversible ErbB2 inhibitors might be able to overcome or prevent the development of potentially key resistance mechanisms such as the gatekeeper secondary T798 mutation.

Next, we defined the comprehensive downstream changes upon lapatinib treatment and only a very select number of genes were altered as early as at 6 hours following ErbB2 blockade. This gene list included a number of highly regulated genes shared by EGFR blockade-induced changes in NSCLC as reported in our previous study, such as cyclin D1, DUSPs, etc corroborating the accuracy of our results and also demonstrating a very prominent overlap in the regulated pathways [26], [27]. We confirmed several of these genes and many are indeed known to participate in key aspects of oncogenic signaling modulation. E.g. cyclin D1 down-regulation results in E2F deactivation with subsequent proliferation arrest and ultimate apoptosis. DUSPs act as natural terminators of MAPK signal transduction and serve as negative feedback mechanisms. DUSP6 expression is regulated by ERK-signaling and exerts anti-tumor effects via negative feedback regulation in NSCLC [26].

Then, we focused on a strongly regulated novel gene, PHLDA1 as it has not been investigated before as a target of oncogenic pathways. This gene shares a pleckstrin-homology (PH) domain with PHLDA3, which was shown to be a direct target gene of p53 and functions as a unique AKT inhibitor through the depletion of membrane-bound phosphatidyl inositols [29], [30]. The exact functions of PHLDA1 are poorly defined. Very little data exists about the expression of PHLDA1 in normal or malignant human tissues. In limited studies, PHLDA1 has been reported to be highly expressed in pancreatic cancers as well as human intestinal tumors, while downregulation of PHLDA1 was noted during melanoma as well as breast cancer progression [31]–[35]. Johnson et al showed that PHLDA1 correlates negatively with estrogen receptor expression and is a strong inhibitor of cell motility, proliferation and transformation in breast cancer cells [36]. PHLDA1 is downregulated in primary breast tumors by immunohistochemistry [36]. However, in that study, there were only 2 ErbB2 positive breast cancer samples, which did not permit strong conclusions. In our study, we find that PHLDA1 is immediately and strongly regulated by ErbB2 signaling. In addition, our in vivo study showed a modest but significant down-regulation of PHLDA1 in primary breast cancer, which is consistent with previously reported findings [31], [36]. We also found that PHLDA1 is significantly less expressed in ErbB2 negative tumors compared with the ErbB2 positive breast cancers. In ErbB2 positive tumors, PHLDA1 protein levels are higher than in ErbB2 negative ones which correlates well with our finding of PHLDA1 expression being induced by ErbB2 signaling. We also demonstrated that PHLDA1 overexpression could inhibit Akt activation, results in reduced cell proliferation and cell motility consistent with its putative function as an Akt pathway inhibitor and suggestive of a candidate tumor suppressor function. These results provide evidence that reduced PHLDA1 expression might be important in breast cancer progression as well as in the modulation of oncogenic ErbB2 signaling and should call for more detailed studies of PHLDA1 expression to assess if it could serve as a useful prognostic marker of disease outcome and whether PHLDA1 expression might be lost in particular subsets of tumors. PHLDA1 overexpression significantly inhibits phosphorylation of AKT that synergizes with lapatinib treatment in SkBr3 and such synergistic effects on cell growth further corroborate a significant role for PHLDA1 in the negative feedback regulation of ErbB2 activity. When the ErbB2 signaling is inhibited by lapatinib, phosphorylation of AKT is downregulated. Consequently, PHLDA1 as the negative feedback factor of ErbB2 pathway is downregulated by ErbB2 signaling inhibition. So PHLDA1 can fine-tune the overall output of ErbB2 signaling in ErbB2 dependent lung and breast cancer cells. We speculate that PHLDA1 might have key inhibitory functions in EGFR and ErbB2-driven lung and breast cancer cells and a better understanding of its functions might point at novel therapeutic options through modulation of this important negative feedback loop.

In summary, our studies define putative and novel resistance mechanisms to lapatinib in ErbB2-positive cancers and identify irreversible EGFR/ErbB2 inhibitors as capable of overcoming such resistance. We also identify a compact set of gene changes upon ErbB2 inhibition and demonstrate strong overlap with EGFR-driven cancers and further identify PHLDA1 as a novel negative feedback inhibitor and candidate tumor suppressor in ErbB2-dependent cancers. Our studies in the aggregate should yield multiple novel ways to expand the benefit of ErbB2 blockade in ErbB2-dependent cancers.

## Supporting Information

Figure S1 **Lapatinib induces cell cycle arrest in SkBr3 cells as detected by BrdU assay.** R1-G1 phase; R2-G2 phase; R3-S phase; R4-G0 phase.
(TIF)

Figure S2 **Dose response of lapatinib and PD168393 on phosphorylation of ErbB2, AKT and ERK in Calu3 cells stimulated with heregulin at 20 ng/ml.**
(TIF)

Figure S3 **Dose-dependent growth inhibition induced by lapatinib or PD168393 treatment for 72 hours in ErbB2-wt and ErbB2-T798I transiently transfected SkBr3 cells as detected by the MTS assay.**
(TIF)

Figure S4 **Lapatinib and PD168393 induce cellular apoptosis in ErbB2-wt and ErbB2-T798I stable Calu3 cell clones following 72 hours of treatment.**
(TIF)

Figure S5 **Quantification of apoptosis assay in SkBr3 cells.**
(TIF)

Figure S6 **Variable PHLDA1 expression in ErbB2 positive and negative cell lines.**
(TIF)

Table S1 **Real-time PCR primers were as follows.**
(DOC)

Table S2 **List of the up-regulated and down-regulated genes comparing lapatinib versus DMSO-treated samples at 6 hours of treatment.**
(DOC)
