# Supplementary material for: Modulation of ErbB2 Blockade in ErbB2-Positive Cancers: The Role of ErbB2 Mutations and PHLDA1
Source: PLoS One. 2014 Sep 19;9(9):e106349. doi: 10.1371/journal.pone.0106349 (PMC4169529; doi:10.1371/journal.pone.0106349)
Supplement: Table S1 — Real-time PCR primers were as follows. (DOC) [file pone.0106349.s007.doc]

**Table S**1

| DUSP6 forward primer | 5’ – CGGAAATGGCGATCAGCAAGACG – 3’ |
| --- | --- |
| DUSP6 reverse primer | 5’ – GCCCCCGAGCCCCAGCACT – 3’ |
| DUSP4 forward primer | 5’ – CGCCTCCCCCTCCGCTCTGC – 3’ |
| DUSP4 reverse primer | 5’ – CGCCGCCGCCATTCTCGTC – 3’ |
| PHLDA1 forward primer | 5’ – AGCGGCGGCCCCTCTTATGC – 3’ |
| PHLDA1 reverse primer | 5’ – CTGCTTCTGCCGCGTGGATTTG – 3’ |
| PHLDA2 forward primer | 5’ – AGCGGCGGCCCCTCTTATGC – 3’ |
| PHLDA2 reverse primer | 5’ – CTGCTTCTGCCGCGTGGATTTG – 3’ |
| CCNG2 forward primer | 5’ – GCTACCCCGGAGAATGATAACACT – 3’ |
| CCNG2 reverse primer | 5’ – AAGGCACAAGGCTAATACAGATGG – 3’ |
| DRE1 forward primer | 5’ – GAGACGCCGGCGCTGGAGAG – 3’ |
| DRE1 reverse primer | 5’ – TCGCTTAGTTGCTGGGGAATCACG – 3’ |
| GAPDH forward primer | 5’ – GCGGGGCTCTCCAGAACATCAT – 3’ |
| GAPDH reverse primer | 5’ – CCAGCCCCAGCGTCAAAGGTG – 3’ |
