# Supplementary material for: Modulation of ErbB2 Blockade in ErbB2-Positive Cancers: The Role of ErbB2 Mutations and PHLDA1
Source: PLoS One. 2014 Sep 19;9(9):e106349. doi: 10.1371/journal.pone.0106349 (PMC4169529; doi:10.1371/journal.pone.0106349)
Supplement: Table S2 — List of the up-regulated and down-regulated genes comparing lapatinib versus DMSO-treated samples at 6 hours of treatment. (DOC) [file pone.0106349.s008.doc]

**Table S2**

| **Gene ID** | **Score(d)** | **Fold Change** | **q-value(%)** | **Gene name** |
| --- | --- | --- | --- | --- |
| 221986_s_at | 51.00782 | 4.95483 | 0 | KLHL24 |
| 211122_s_at | 46.38183 | 4.851524 | 0 | CXCL11 |
| 209102_s_at | 44.88962 | 2.811998 | 0 | HBP1 |
| 215001_s_at | 44.21148 | 2.080593 | 0 | GLUL |
| 203588_s_at | 36.17039 | 2.197197 | 0 | TFDP2 |
| 221985_at | 33.15313 | 4.220604 | 0 | KLHL24 |
| 205027_s_at | 32.74423 | 3.351044 | 0 | MAP3K8 |
| 204491_at | 32.54785 | 3.69724 | 0 | PDE4D |
| 209864_at | 28.52365 | 2.922762 | 0 | FRAT2 |
| 212135_s_at | 27.81067 | 2.003486 | 0 | ATP2B4 |
| 212675_s_at | 25.27888 | 2.903494 | 0 | Transcribed locus |
| 204917_s_at | 21.78953 | 2.463919 | 0 | MLLT3 |
| 218285_s_at | 21.57465 | 2.488543 | 0 | BDH2 |
| 217122_s_at | 20.75915 | 2.105742 | 0 | SLC35E2 |
| 205870_at | 20.42029 | 8.230281 | 0 | BDKRB2 |
| 201009_s_at | 19.98998 | 6.841295 | 0 | TXNIP |
| 205518_s_at | 19.97706 | 2.79091 | 0 | CMAH |
| 203640_at | 18.75815 | 2.279454 | 0 | MBNL2 |
| 203632_s_at | 17.69108 | 3.275462 | 0 | GPRC5B |
| 221756_at | 17.61811 | 3.283974 | 0 | MGC17330 |
| 218631_at | 15.78988 | 3.479511 | 0 | PI4K2A |
| 209568_s_at | 15.46642 | 3.111932 | 0 | RGL1 |
| 218692_at | 15.4132 | 2.427374 | 0 | GOLSYN |
| 201010_s_at | 15.31917 | 7.991968 | 0 | TXNIP |
| 203080_s_at | 15.23171 | 2.673902 | 0 | BAZ2B |
| 213624_at | 14.87215 | 2.106818 | 0 | SMPDL3A |
| 202227_s_at | 14.68114 | 2.344304 | 0 | BRD8 |
| 218031_s_at | 14.62323 | 2.150599 | 0 | FOXN3 |
| 203043_at | 14.54477 | 2.92925 | 0 | DHRSX |
| 209431_s_at | 14.38536 | 2.102162 | 0 | PATZ1 |
| 217906_at | 14.1091 | 2.106567 | 0 | KLHDC2 |
| 218806_s_at | 14.1074 | 4.813903 | 0 | VAV3 |
| 201384_s_at | 13.7148 | 2.073093 | 0 | NBR1 |
| 210761_s_at | 13.6683 | 2.526687 | 0 | GRB7 |
| 203589_s_at | 13.66703 | 2.906036 | 0 | TFDP2 |
| 203963_at | 13.61142 | 2.609091 | 0 | CA12 |
| 211778_s_at | 13.50539 | 3.356408 | 0 | OVOL2 |
| 211559_s_at | 13.49955 | 2.47516 | 0 | CCNG2 |
| 204820_s_at | 13.45611 | 3.202365 | 0 | BTN3A3 member A2 |
| 220915_s_at | 13.39426 | 2.941603 | 0 | LOC442367 |
| 209760_at | 13.31947 | 2.316553 | 0 | KIAA0922 |
| 219344_at | 13.21577 | 2.155429 | 0 | SLC29A3 |
| 222361_at | 13.15135 | 2.197961 | 0 | LOC643224 |
| 202364_at | 13.06033 | 2.401586 | 0 | MXI1 |
| 218807_at | 13.05742 | 5.048035 | 0 | VAV3 |
| 205486_at | 13.02436 | 2.128392 | 0 | TESK2 |
| 201008_s_at | 12.73689 | 6.306225 | 0 | TXNIP |
| 217127_at | 12.36817 | 2.260616 | 0 | CTH |
| 218268_at | 12.33765 | 2.085547 | 0 | TBC1D15 |
| 209479_at | 11.76778 | 2.616831 | 0 | CCDC28A |
| 206757_at | 11.75769 | 2.098748 | 0 | PDE5A |
| 209230_s_at | 11.67866 | 3.053588 | 0 | NUPR1 |
| 212151_at | 11.65986 | 2.296848 | 0 | PBX1 |
| 202181_at | 11.57391 | 2.379845 | 0 | KIAA0247 |
| 213106_at | 11.51992 | 2.671788 | 0 | ATP8A1 |
| 219566_at | 11.42254 | 2.088869 | 0 | PLEKHF1 |
| 210163_at | 11.40748 | 2.559547 | 0 | CXCL11 |
| 200648_s_at | 10.95671 | 2.756065 | 0 | GLUL |
| 218491_s_at | 10.85379 | 2.195283 | 0 | THYN1 |
| 211732_x_at | 10.61277 | 2.63143 | 0 | HNMT |
| 217028_at | 10.57974 | 3.180583 | 0 | CXCR4 |
| 212148_at | 10.52727 | 2.661253 | 0 | PBX1 |
| 215867_x_at | 10.49896 | 2.447556 | 0 | CA12 |
| 203973_s_at | 10.24618 | 2.464767 | 0 | KIAA0146 |
| 212677_s_at | 10.04716 | 2.932432 | 0 | CEP68 |
| 209750_at | 9.985347 | 2.242041 | 0 | NR1D2 |
| 219049_at | 9.952315 | 3.12605 | 0 | ChGn |
| 203140_at | 9.881804 | 2.009871 | 0 | BCL6 |
| 214775_at | 9.840514 | 2.991007 | 0 | N4BP3 |
| 208763_s_at | 9.745831 | 4.902036 | 0 | TSC22D3 |
| 204821_at | 9.735951 | 2.284479 | 0 | BTN3A3 |
| 219164_s_at | 9.477538 | 2.188985 | 0 | ATG2B |
| 202769_at | 9.360382 | 2.013582 | 0 | CCNG2 |
| 208892_s_at | -85.3023 | 0.126178 | 0 | DUSP6 |
| 218000_s_at | -80.6382 | 0.297667 | 0 | PHLDA1 |
| 208712_at | -57.6781 | 0.150877 | 0 | CCND1 |
| 203692_s_at | -52.2974 | 0.430391 | 0 | E2F3 |
| 201196_s_at | -50.458 | 0.411272 | 0 | AMD1 |
| 219821_s_at | -49.8218 | 0.250179 | 0 | GFOD1 |
| 201041_s_at | -48.0033 | 0.14947 | 0 | DUSP1 |
| 218647_s_at | -46.652 | 0.404876 | 0 | MANEAL |
| 205476_at | -43.5315 | 0.286911 | 0 | CCL20 |
| 204363_at | -41.9482 | 0.311877 | 0 | F3 |
| 201341_at | -40.6365 | 0.20472 | 0 | ENC1 |
| 203044_at | -38.2794 | 0.405714 | 0 | CHSY1 |
| 210017_at | -35.2517 | 0.470468 | 0 | MALT1 |
| 217996_at | -34.853 | 0.072108 | 0 | PHLDA1 |
| 201465_s_at | -34.4836 | 0.484878 | 0 | JUN |
| 209210_s_at | -32.9563 | 0.414887 | 0 | PLEKHC1 |
| 219474_at | -32.8438 | 0.30911 | 0 | C3orf52 |
| 217173_s_at | -30.6609 | 0.496489 | 0 | LDLR |
| 213927_at | -29.7597 | 0.264087 | 0 | MAP3K9 |
| 205681_at | -27.8672 | 0.251349 | 0 | BCL2A1 |
| 214696_at | -26.6834 | 0.474607 | 0 | MGC14376 |
| 204011_at | -26.5134 | 0.281097 | 0 | SPRY2 |
| 204958_at | -25.4089 | 0.361947 | 0 | PLK3 |
| 201110_s_at | -23.6469 | 0.289312 | 0 | THBS1 |
| 202668_at | -23.2504 | 0.187574 | 0 | EFNB2 |
| 217997_at | -22.8359 | 0.041821 | 0 | PHLDA1 |
| 202693_s_at | -22.3836 | 0.368347 | 0 | STK17A |
| 219500_at | -22.2801 | 0.39777 | 0 | CLCF1 |
| 212845_at | -21.9402 | 0.41745 | 0 | SAMD4A |
| 205490_x_at | -21.4028 | 0.394275 | 0 | GJB3 |
| 212723_at | -21.2925 | 0.499466 | 0 | JMJD6 |
| 203072_at | -20.8014 | 0.410051 | 0 | MYO1E |
| 210117_at | -20.4697 | 0.364765 | 0 | SPAG1 |
| 201197_at | -20.1534 | 0.4864 | 0 | AMD1 |
| 202284_s_at | -19.7894 | 0.324152 | 0 | CDKN1A |
| 208937_s_at | -19.7297 | 0.165841 | 0 | ID1 |
| 218963_s_at | -19.5818 | 0.373927 | 0 | KRT23 |
| 213618_at | -19.4409 | 0.368366 | 0 | CENTD1 |
| 214141_x_at | -19.2923 | 0.416568 | 0 | SFRS7 |
| 218886_at | -19.2695 | 0.438458 | 0 | PAK1IP1 |
| 203693_s_at | -19.1594 | 0.406847 | 0 | E2F3 |
| 201693_s_at | -19.1201 | 0.148276 | 0 | EGR1 |
| 221009_s_at | -18.8786 | 0.100785 | 0 | ANGPTL4 |
| 213506_at | -18.5872 | 0.472924 | 0 | F2RL1 |
| 201694_s_at | -18.5417 | 0.116829 | 0 | EGR1 |
| 201109_s_at | -18.4665 | 0.297538 | 0 | THBS1 |
| 202613_at | -18.3528 | 0.41603 | 0 | CTPS |
| 204435_at | -18.2768 | 0.493908 | 0 | NUPL1 |
| 212233_at | -18.1537 | 0.385509 | 0 | 3'UTR of hypothetical protein (ORF1) |
| 217998_at | -16.9678 | 0.375945 | 0 | PHLDA1 |
| 204897_at | -16.9354 | 0.192756 | 0 | PTGER4 |
| 216061_x_at | -16.7599 | 0.365442 | 0 | PDGFB |
| 214599_at | -16.4738 | 0.427429 | 0 | IVL |
| 205891_at | -16.0199 | 0.488299 | 0 | ADORA2B |
| 202270_at | -15.9686 | 0.341212 | 0 | GBP1 |
| 201340_s_at | -15.6715 | 0.150138 | 0 | ENC1 |
| 221020_s_at | -15.6289 | 0.480768 | 0 | SLC25A32 |
| 204014_at | -15.619 | 0.38073 | 0 | DUSP4 |
| 33323_r_at | -15.5926 | 0.345943 | 0 | SFN |
| 204420_at | -15.1042 | 0.112585 | 0 | FOSL1 |
| 218889_at | -15.0961 | 0.458778 | 0 | NOC3L |
| 213680_at | -15.0162 | 0.438218 | 0 | KRT6B |
| 209893_s_at | -14.897 | 0.457029 | 0 | FUT4 |
| 209909_s_at | -14.6298 | 0.420381 | 0 | M19154 |
| 209101_at | -14.506 | 0.298068 | 0 | CTGF |
| AFFX-M27830_5_at | -14.1519 | 0.386827 | 0 | AFFX-M27830_5 |
| 209908_s_at | -13.8478 | 0.428765 | 0 | TGFB2 |
| 204030_s_at | -13.7223 | 0.425701 | 0 | SCHIP1 |
| 204094_s_at | -13.6345 | 0.363181 | 0 | TSC22D2 |
| 203499_at | -13.5974 | 0.125657 | 0 | EPHA2 |
| 209291_at | -13.5507 | 0.298605 | 0 | ID4 |
| 209457_at | -13.0732 | 0.460962 | 0 | DUSP5 |
| 221750_at | -13.0256 | 0.348936 | 0 | HMGCS1 |
| 218368_s_at | -12.9706 | 0.247828 | 0 | TNFRSF12A |
| 214212_x_at | -12.7679 | 0.404378 | 0 | PLEKHC1 |
| AFFX-M27830_M_at | -12.6708 | 0.448594 | 0 | AFFX-M27830_M |
| 210026_s_at | -12.6512 | 0.407199 | 0 | CARD10 |
| 206662_at | -12.6153 | 0.486309 | 0 | GLRX |
| 204597_x_at | -12.5005 | 0.315469 | 0 | STC1 |
| 201129_at | -12.4373 | 0.429533 | 0 | SFRS7 |
| 208711_s_at | -12.3951 | 0.187663 | 0 | CCND1 |
| 219099_at | -12.3661 | 0.393893 | 0 | C12orf5 |
| 207213_s_at | -12.1565 | 0.362195 | 0 | USP2 |
| 202669_s_at | -12.0371 | 0.202061 | 0 | EFNB2 |
| 221489_s_at | -12.0227 | 0.253328 | 0 | SPRY4 |
| 208893_s_at | -11.9298 | 0.263979 | 0 | DUSP6 |
| 202269_x_at | -11.722 | 0.356825 | 0 | GBP1 |
| 209803_s_at | -11.6321 | 0.125931 | 0 | PHLDA2 |
| 204595_s_at | -11.5376 | 0.278548 | 0 | STC1 |
| 201464_x_at | -11.341 | 0.491798 | 0 | JUN |
| 202643_s_at | -10.8927 | 0.474896 | 0 | TNFAIP3 |
| 215243_s_at | -10.8831 | 0.3983 | 0 | GJB3 |
| 208112_x_at | -10.8488 | 0.491736 | 0 | EHD1 |
| 202644_s_at | -10.767 | 0.489569 | 0 | TNFAIP3 |
| 211924_s_at | -10.5616 | 0.320129 | 0 | PLAUR |
| 209209_s_at | -10.542 | 0.423085 | 0 | PLEKHC1 |
| 210845_s_at | -10.4492 | 0.32268 | 0 | PLAUR |
| 219496_at | -10.4492 | 0.419421 | 0 | ANKRD57 |
| 202464_s_at | -10.394 | 0.394713 | 0 | PFKFB3 |
| 201631_s_at | -10.3247 | 0.304776 | 0 | IER3 |
| 209286_at | -10.1626 | 0.467305 | 0 | CDC42EP3 |
| 209276_s_at | -10.0898 | 0.412102 | 0 | GLRX |
| 205899_at | -10.0787 | 0.413458 | 0 | CCNA1 |
| 202859_x_at | -9.96414 | 0.492113 | 0 | IL8 |
| 209892_at | -9.72373 | 0.397315 | 0 | FUT4 |
| 219503_s_at | -9.70562 | 0.415035 | 0 | TMEM40 |
| 213358_at | -9.60541 | 0.453318 | 0 | LOC647277 |
| 212803_at | -9.2734 | 0.492277 | 0 | NAB2 |
| 218182_s_at | -9.22901 | 0.460513 | 0 | CLDN1 |
| 200808_s_at | -9.07176 | 0.478149 | 0 | ZYX |
